# The Role of TRPV4 in Regulating Innate Immune Cell Function in Lung Inflammation

**DOI:** 10.3389/fimmu.2020.01211

**Published:** 2020-06-26

**Authors:** Rachel G. Scheraga, Brian D. Southern, Lisa M. Grove, Mitchell A. Olman

**Affiliations:** ^1^Respiratory Institute, Cleveland Clinic, Cleveland, OH, United States; ^2^Department of Inflammation and Immunity, Lerner Research Institute, Cleveland Clinic, Cleveland, OH, United States

**Keywords:** TRPV4 (transient receptor potential vanilloid-4), macrophage, innate immunity, lung inflammation and injury, MAPK

## Abstract

Ion channels/pumps are essential regulators of innate immune cell function. Macrophages have been increasingly recognized to have phenotypic plasticity and location-specific functions in the lung. Transient receptor potential vanilloid 4 (TRPV4) function in lung injury has been shown to be stimulus- and cell-type specific. In the current review, we discuss the importance of TRPV4 in macrophages and its role in phagocytosis and cytokine secretion in acute lung injury/acute respiratory distress syndrome (ARDS). Furthermore, TRPV4 controls a MAPK molecular switch from predominately c-Jun N-terminal kinase, JNK activation, to that of p38 activation, that mediates phagocytosis and cytokine secretion in a matrix stiffness-dependent manner. Expanding knowledge regarding the downstream mechanisms by which TRPV4 acts to tailor macrophage function in pulmonary inflammatory diseases will allow for formulation of novel therapeutics.

## Introduction

Ion channels and transporters are rapidly being recognized as essential for basic physiological functions of immune cells (1, 2). However, gaps in knowledge remain on the intracellular molecular mechanisms by which ion channels contribute to immune cell function. Calcium and other cations (such as sodium and potassium) have been shown to act as second messengers to regulate innate immune cell function and activation (3). For example, macrophage migration, polarization, phagocytosis, and cytokine secretion have been shown to be regulated through calcium (4–6). One such mechanism of calcium regulation in the cell is through the calcium permeable cation channel transient receptor potential vanilloid 4 (TRPV4). TRPV4 is a mechanosensitive cation channel that is essential for macrophage activation functions such as macrophage phagocytosis and cytokine secretion in a matrix stiffness-dependent manner (7, 8). The current review focuses on experimental data illustrating the importance of TRPV4 on immune cell function. We appreciate all the important contributions to the literature in this field, however given space considerations we have focused on what is perceived to be directly relevant to this review.

## Macrophage Heterogeneity in the Lung

The lung is constantly exposed to inhaled particles and pathogens from the environment (9). Hence, lung innate immunity needs to be tightly regulated and phenotypically plastic in order to simultaneously maintain homeostasis and clear foreign invaders (10). Recently published data characterize macrophage phenotypic subsets based on their location in the lung [alveolar (AM) vs. interstitial (IM)] in naïve and injured (LPS/bleomycin treatment) conditions (11–13). The alveolar and interstitial subsets have been further divided based on site of origin into “resident” and “recruited” macrophage populations after injury or inflammation, by fate mapping, and lineage tracing models (11, 14–16). “Resident” macrophages populate the lung a few days after birth from fetal monocytes, and self-renew after injury (14, 15, 17). In contrast, recruited lung macrophages populate the lung only after injury and are derived from circulating monocytes that originated from the bone marrow (14, 15, 18). Despite this anatomic lineage and genomic classification, the *in vivo* biologic functions of both resident/recruited alveolar and interstitial macrophage populations are not fully understood. The microenvironment plays a key role in reprograming the monocyte/macrophage phenotypic response to lung injury (19, 20). Reprograming of the macrophage phenotype is not as simple as the classically-defined *in vitro* M1/M2 paradigm characterized by surface marker labeling (21–24). The molecular pathways *in vivo* by which the macrophage phenotype and function change in response to the microenvironment have yet to be fully described.

## Macrophage Function and Signaling in Acute Lung Injury

Other literature has extensively characterized the important macrophage mediated mechanisms of chronic lung injury. Herein, this review will focus on the role of macrophages in acute lung injury. Macrophages are the most abundant, and critical cells, that maintain homeostasis in the lung (9). Macrophages have also been shown to play an important role in orchestrating the acute lung injury and repair process (25). Acute lung injury, both from non-infectious and infectious inflammation is a complex process. Acute lung injury is a consequence of endothelial and/or alveolar epithelial injury, followed by recruitment, and accumulation of inflammatory cells in the injured/stiffened alveolus (26–28). Macrophages have surface receptors that recognize pathogen (PAMPs) and/or damage-associated molecular patterns (DAMPs) to recruit inflammatory cells (e.g., neutrophils, recruited alveolar/interstitial macrophages) and coordinate both activation and cessation of inflammation (29). Macrophages function to phagocytize invading organisms, apoptotic cells/neutrophils, or particles. In addition, macrophages secrete, and respond to pro- and anti-inflammatory cytokines and chemokines (e.g., IL-1β, TNF-α, IL-8, IL-6, IL-10) (30–34). Activation of macrophages in response to infection occurs in part through coordination of activation of key stress activated pathways including Nuclear factor kappa-light-chain-enhancer of activated B cells (NF-κB), Interferon regulatory factor 3 (IRF3), Stimulator of interferon genes (STING), and Mitogen activated protein kinases (MAPKs) (35). The MAPK family (i.e., p38, ERK, and JNK) exhibits functional cross-talk and redundant functions in inflammation (35). p38 and JNK have been shown to be activated in lung injury in response to infection and to be important in macrophage activation functions such as phagocytosis (36). Control of persistent activation of MAPKs is mainly regulated by the phosphatases, dual-specificity serine threonine phosphatases/MAPK phosphatases (DUSPs/MKPs) (37, 38). It has been described that increased lung stiffness regulates MAPK/phosphatase cross-talk, while others have shown that alveolar vessel wall stiffness increases >10-fold (from 3 to 45 kPa) after intratracheal LPS-induced lung injury in mice (39), thus providing mechanistic insight into how macrophages can respond to cues from the injured lung. However, distinct mechanisms whereby increased lung tissue stiffness/injury controls the MAPK and phosphatase cross-talk is poorly understood.

## The TRPV4 Channel and Mechanobiology Of the Lung

Transient receptor potential (TRP) channels are a family of 6 transmembrane domain proteins that are permeable to multiple cations including calcium (40). TRP channels are widely expressed in multiple tissues and cell-types with varied physiologic functions (40). Specifically, the TRP family member, TRPV4, is a ubiquitously-expressed, plasma membrane-based, calcium-permeable channel that is sensitized and activated by both chemical [5,6-Epoxyeicosatrienoic acid (EET), 4 alpha-phorbol 12,13-didecanoate (4-αPDD)] and physical stimuli (temperature, stretch, and hypotonicity) (40–43). TRPV4 can initiate intracellular, celltype and context-specific signals that depend on local increases in intracellular calcium which could act as a second messenger, and/or induce heterodimerization with other channels, activate kinases, and/or directly interact with cytoskeletal proteins via intracellular amino-(NH_2_) and carboxy-(COOH) terminal tails (44–47).

It has been increasingly recognized that cellular responses depend on the biophysical properties of the surrounding lung tissue environment (48, 49). Thus, mechanical cues from lung tissue stretch/stiffness can alter cellular responses to soluble mediators (e.g., growth factors, cytokines, chemokines) resulting in cellular dysfunction and disease. The mechanosensitive channel, TRPV4 has been implicated in mouse models of lung injury/fibrosis, which include hydrochloric acid, pulmonary edema, ventilator-associated lung parenchymal overdistension, and from our group, pulmonary fibrosis (50–53). The recent mini-review by Michalick and Kuebler in Frontiers Immunology further supports the concept that TRPV4 may connect mechanosensation to immunity in the lung (54). Given TRPV4's published role on regulating activity and infectivity of RNA viruses such as Zika, it remains possible that TRPV4 plays a role in the profound lung injury observed in the current SARS-CoV-2 pandemic (55). Conflicting data exist on the role of TRPV4 in mouse models of lung inflammation/injury, which seem to depend on the inciting agent, mechanism of injury, and the effector cell type (50–53). In ventilator-induced lung injury, macrophage TRPV4 has shown to exacerbate the lung injury (51, 52). Similarly in acid-induced lung injury, TRPV4 also exacerbates the lung injury (53). Furthermore, a recent study using a single pharmacologic inhibitor of TRPV4 revealed decreased lung injury after intratracheal instillation of LPS for 24 h (56). Our data, in a clinically relevant infectious model of lung injury, support the hypothesis that TRPV4 is protective from injury (7). In support of our findings, epithelial cell TRPV4 similarly protects the lung, but in a somewhat distinct, rapid direct LPS-induced lung injury model (3 h) (57). Despite the conflicting data on the role of TRPV4 in mouse models of lung injury, some consensus exists on the importance of TRPV4 in macrophage signaling (7, 8, 52). Further understanding of the molecular mechanisms by which macrophage TRPV4 is involved in the pathogenesis of lung injury will allow for a therapeutic target to ameliorate lung injury.

## TRPV4 in Macrophages and Lung Injury

The calcium ion channel, TRPV4 is an essential mechanosensor that is required for effective phagocytosis *in vitro* and protects against infection-associated lung injury *in vivo* (7, 8). TRPV4 in macrophages have been shown to exacerbate ventilator-associated lung injury, and macrophages are key effector cells in the lung injury process (51, 52). Calcium has long been described as a mediator of many discrete steps in the phagocytic process (58). In addition, effective phagocytosis requires cytoskeletal rearrangements and direct interaction with the biophysical properties of the matrix (19, 20, 59). However, the key regulatory ion channels/pumps by which calcium influx into the cell is controlled during phagocytosis is not fully elucidated.

Published work first revealed that differentiated murine bone marrow-derived macrophages (BMDMs) express equal amounts of TRPV4 that was functionally active with or without LPS. TRPV4 in macrophages functions to effectively phagocytize both non-opsonized, *E. coli* bacteria, and opsonized (FcR-dependent), IgG-coated latex beads, *in vitro* in response to LPS (8). The LPS-stimulated phagocytic response was induced, in our hands, under conditions of pathophysiologic-range extracellular matrix stiffnesses, in the range noted in inflamed or injured lung (≥8–25 kPa) (16). TRPV4 had no effect on basal phagocytosis. In addition, TRPV4 downregulated LPS-induced IL-1β secretion and upregulated IL-10 secretion. Further, this TRPV4 mediated anti-inflammatory cytokine profile (↓ IL-1β, ↑ IL-10) was dependent on pathophysiologic-range matrix stiffness. To apply *in vivo* relevance, TRPV4 was found to be required for effective alveolar macrophage phagocytosis of IgG-coated latex beads in live mice *in vivo* (8). Taken together, TRPV4 is necessary for effective opsonized and non-opsonized macrophage phagocytosis and an anti-inflammatory cytokine profile, in a stiffness-dependent manner *in vitro* and *in vivo* (8).

To expand on this work, TRPV4's *in vivo* relevance to human disease, and molecular mechanism by which TRPV4 mediates its phagocytic and cytokine secretory effects was determined. TRPV4 was found to function to protect the lung from injury in an experimental model of *Pseudomonas aeruginosa* pneumonia in intact mice (7). Lung injury was measured by (i) inflammatory cell infiltration, (ii) total protein in whole lung lavage, (iii) cytokine secretion, and (iv) lung parenchymal consolidation. In addition, TRPV4 was required for effective clearance of the *P. aeruginosa* bacteria as measured by colony forming units retained in the lung in WT, as compared to global TRPV4 KO mice. Next, macrophages were identified as the critical cell type required to clear the *P. aeruginosa* infection by flow cytometric techniques (7).

**Figure 1:**
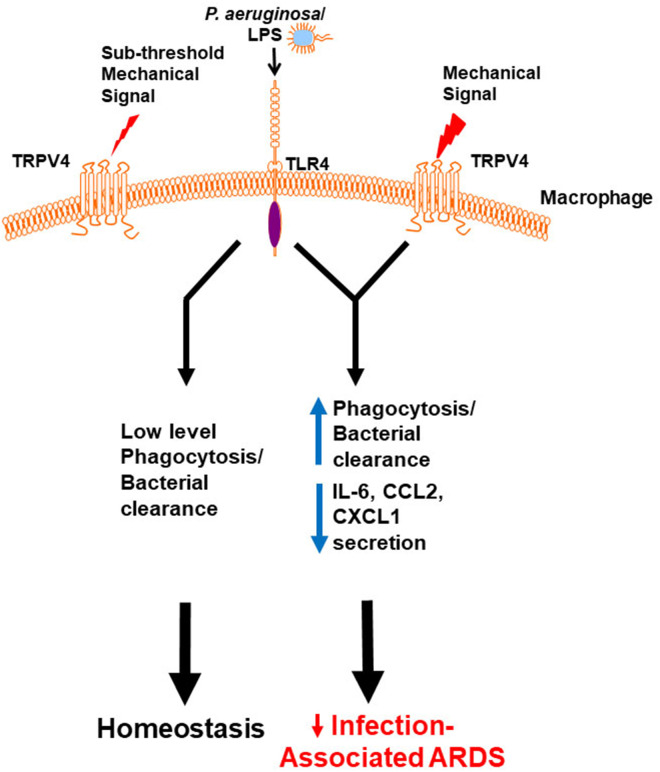
Working model demonstrates that a mechanical signal through TRPV4 regulates the LPS response. Our data shows in the **absence** of an above threshold mechanical signal, TRPV4 does not influence the minimal LPS/TLR4 response, leading to low level phagocytosis/bacterial clearance, and resultant lung homeostasis. In the **presence** of an above threshold mechanical signal, TRPV4 modulates with the LPS/TLR4 response to increase phagocytosis and decrease pro-inflammatory cytokine secretion, thereby protecting the lung from infection-associated injury/ARDS (7). Adapted from original publication in *The Journal of Immunology*. Copyright^©^ 2020 The American Association of Immunologists, Inc.

To determine the molecular mechanism by which TRPV4 protects the lung from injury and clears bacteria, putative intracellular signaling pathways were investigated that are known to regulate LPS signals in macrophages (60). TRPV4 controlled molecular switching from predominate JNK activation to that of p38, in a stiffness-dependent manner. Since MAPK phosphorylation occurs commonly through the phosphatase family DUSPs/MKPs, it was postulated that DUSPs controlled the TRPV4-mediated MAPK molecular switch. TRPV4 acted to increase DUSP1 and then functioned to selectively dephosphorylate/deactivate JNK. Hence, the TRPV4-mediated MAPK molecular switch was found to be controlled through DUSP1 in a stiffness-dependent manner. TRPV4 additionally enhanced p38 activation thereby driving effective phagocytosis, while inhibiting JNK thereby decreasing pro-inflammatory cytokine secretion (IL-6, CCL2, and CXCL1). Finally, TRPV4 is also required for macrophage phagocytosis and p38 activation in healthy human monocyte-derived macrophages. Taken together, published work shows that TRPV4 in macrophages protected the lung from infection-associated lung injury through regulation of MAPK activation switching via DUSP1 (7). TRPV4 provides a novel mechanistic link between the mechanoenvironmental properties of the lung and innate immune cell function (Figure 1).

## Important Future Directions

Ongoing questions remain regarding the molecular mechanism by which TRPV4 activity is regulated or how TRPV4 is directly activated. In addition, the molecular signals by which TRPV4 regulates the MAPK molecular switch remain unknown. Since TRPV4 can directly interact with signaling molecules via its amino (NH_2_) and carboxy (COOH) terminal intracellular tails, the key signaling molecules that interact with TRPV4 to enhance macrophage phagocytosis and limit cytokine secretion is an active area of investigation. It remains possible that the TRPV4 interacting partners are not TLR4-dependent, as other data suggests that TRPV4 interacts with PI3K to mediate pulmonary fibrosis (61). The role of TRPV4 in different macrophage populations after infection remains an important question. Other cation/calcium channels (e.g., Piezo) have been shown to have an effect on immune cells in a mechanosensitive manner and interestingly recent work demonstrates TRPV4 is required for Piezo1-induced pancreatitis (62, 63). The molecular pathways *in vivo* by which the macrophage phenotype and function change in response to the microenvironment have yet to be fully described. For example, it will also be interesting in the future to explore TRPV4's action in response to (a) other types of infectious stimuli (e.g., Gram positive organisms, viral infections such as SARS-CoV-2), (b) sex differences, and (c) transcriptional/epigenetic mechanisms. An important goal of future work is to integrate mouse and human macrophage experiments, however there is limitation to this approach. It is well-known that mouse models do not fully recapitulate human disease, and the mouse immune system is programmed differently than that of humans (64). In order to circumvent this limitation, investigators utilize human diseased tissue which may provide insight into disease mechanisms, however it is usually in an *in vitro* setting. Hence, data obtained from mouse models and human disease tissue have their own independent strengths. Therefore, it is important to interpret the findings in a contextual nature to determine the relevance to mechanisms of human disease and design targeted pharmacologic therapies.

## Conclusion

In summary, macrophages function in the lung to maintain homeostasis and clear environmental particles and pathogens. Extensive macrophage heterogeneity and plasticity allows for fine-tuning of the inflammatory response upon inflammation or infection. Ion channels have been shown to play a key role in regulating innate immune function and contribute to the pathogenesis of inflammatory/infectious lung diseases. The cation channel TRPV4 has been implicated in lung diseases associated with parenchymal stretch and inflammation or infection. The data outlined in this review show the importance of macrophage TRPV4 in response to infection and lung injury. The data shows that TRPV4 is **(a)** functionally active in macrophages, **(b)** required for effective non-opsonized and opsonized phagocytosis *in vitro* and *in vivo*, and **(c)** required for secretion of an anti-inflammatory cytokine profile by macrophages. These phagocytic and cytokine effects in macrophages were both dependent on matrix stiffness in the range of injured or fibrotic lung (8). In addition, TRPV4 **(a)** protects the lung from injury after *P. aeruginosa* pneumonia, **(b)** mediates the lung injury effects through MAPK molecular switching, and **(c)** is required for effective macrophage phagocytosis in human macrophages. This MAPK switching effect in macrophages was also dependent on matrix stiffness in the range of injured or fibrotic lung (7). Collectively, TRPV4 is shown to play a novel role in protecting the lung from infection-associated lung injury by regulating the phagocytic and cytokine secretory response to infection, and therefore may be a potential therapeutic target in the pathogenesis of acute lung injury.

## Author Contributions

RS, BS, LG, and MO reviewed the literature and wrote the paper. All authors contributed to the article and approved the submitted version.

## Conflict of Interest

The authors declare that the research was conducted in the absence of any commercial or financial relationships that could be construed as a potential conflict of interest.
